# Interaction between variants in the CYP2C9 and POR genes and the risk of sulfonylurea‐induced hypoglycaemia: A GoDARTS Study

**DOI:** 10.1111/dom.13046

**Published:** 2017-08-25

**Authors:** Tanja Dujic, Kaixin Zhou, Louise A. Donnelly, Graham Leese, Colin N. A. Palmer, Ewan R. Pearson

**Affiliations:** ^1^ Department of Biochemistry and Clinical Analysis, Faculty of Pharmacy University of Sarajevo Sarajevo Bosnia and Herzegovina; ^2^ Division of Molecular and Clinical Medicine, School of Medicine University of Dundee Dundee Scotland, UK; ^3^ Department of Endocrinology and Diabetes, Ninewells Hospital and Medical School University of Dundee Dundee Scotland, UK

**Keywords:** hypoglycaemia, pharmacogenetics, sulphonylureas, type 2 diabetes

## Abstract

Data on the association of CYP2C9 genetic polymorphisms with sulfonylurea (SU)‐induced hypoglycaemia (SH) are inconsistent. Recent studies showed that variants in the P450 oxidoreductase (POR) gene could affect CYP2C9 activity. In this study, we explored the effects of POR*28 and combined CYP2C9*2 and CYP2C9*3 genotypes on SH and the efficacy of SU treatment in type 2 diabetes. A total of 1770 patients were included in the analysis of SU efficacy, assessed as the combined outcome of the HbA1c reduction and the prescribed SU daily dose. Sixty‐nine patients with severe SH were compared with 311 control patients. The number of CYP2C9 deficient alleles was associated with nearly three‐fold higher odds of hypoglycaemia (OR, 2.81; 95% CI, 1.30‐6.09; P = .009) and better response to SU treatment (β, −0.218; SE, 0.074; P = .003) only in patients carrying the POR*1/*1 genotype. Our results indicate that interaction between CYP2C9 and POR genes may be an important determinant of efficacy and severe adverse effects of SU treatment.

## 1 INTRODUCTION

Despite development of novel pharmacological agents, sulfonylureas (SUs) continue to be a cornerstone in type 2 diabetes (T2D) treatment. The most common and potentially most serious adverse effect of sulfonylurea therapy is hypoglycaemia, which limits their use.^1^ Severe hypoglycaemia may result in significant morbidity, including higher risk of dementia, stroke and mortality.^2^, ^3^, ^4^ In addition to established clinical factors, identification of genetic factors which can increase the risk of hypoglycaemia can contribute to safer treatment with SU agents.

SUs are metabolized in the liver primarily by the CYP2C9 enzyme.^5^ CYP2C9 is highly polymorphic, with CYP2C9*1 as a major allele. The 2 most common variants, CYP2C9*2 (R144C, rs1799853) and CYP2C9*3 (I359L, rs1057910), have been associated with impaired function and poor metabolism phenotypes^6^ (Table S1). The impact of *2 and *3 polymorphisms on the pharmacokinetics of SUs has been demonstrated in studies in healthy subjects.^7^ In the largest study to date on the effect of *CYP2C9* variants on therapeutic response to SUs, we showed an association of CYP2C9*2 and CYP2C9*3 alleles with greater glycaemic response to SUs and a lower rate of treatment failure,^8^ confirming earlier pharmacokinetic data. In line with this, in the Rotterdam study, patients with T2D carrying the CYP2C9*3 allele required a lower dose of tolbutamide to regulate glycaemia, compared to patients with the wild‐type *CYP2C9* genotype.^9^

Despite these findings, limited data from small‐sized studies have not provided convincing evidence of the association between *CYP2C9* poor‐metabolizer genotypes and the risk of SU‐induced hypoglycaemia.^10^ A small study recently showed that a common polymorphism in the gene encoding a CYP450 redox partner, enzyme P450 oxidoreductase (POR), POR*28 (A503V, rs1057868), could mask the effect of *CYP2C9* variants on the risk of hypoglycaemia associated with SU treatment.^11^ POR transfers electrons from NADPH to the CYP450 enzymes and is essential for their activity. The common POR*28 variant showed modest increase in CYP2C9‐mediated metabolism of 3 different model substrates in an in vitro study.^12^ In line with this, the effect of *CYP2C9* reduced‐function variants on CYP2C9 activity may manifest only in combination with the POR*1/*1 genotype.^11^

In this study, we aimed to assess the impact of CYP2C9*2, CYP2C9*3 and POR*28 alleles, and their interaction, on SU‐induced hypoglycaemia in patients with T2D. Furthermore, we explored the association of the *CYP2C9* and *POR* variants with the efficacy of SU treatment in T2D.

## 2 METHODS

We used the Scottish Ambulance Service (SAS) data and the Accident and Emergency (A and E) diagnosis records to identify cases of severe hypoglycaemia in Tayside and Fife, Scotland. These data were linked to the Genetics of Diabetes Audit and Research Tayside Study (GoDARTS) database, which contains genetic information and longitudinal biochemistry records, prescriptions and other clinical data in an anonymized form, on nearly 10 000 patients with T2D in Tayside, from 1992 onwards. The GoDARTS study was approved by the Tayside Medical Ethics Committee. Informed consent was obtained for all participants. The use of the GoDARTS bioresource for the study of diabetes pharmacogenetics was approved by the Tayside Tissue Bank.

We identified a total of 339 patients with T2D who were under treatment with SUs, and had experienced a hypoglycaemic event requiring medical assistance in the period 2008 to 2015. Among these, there were 69 patients who were in the GoDARTS study and for whom genetic data were available. Between 1 and 5 controls were selected for each case from the patients with T2D who had experienced no hypoglycaemic events and were treated with SUs during the same time period. These controls were matched for age, sex and age of T2D diagnosis. Patients treated with insulin were excluded. The date of the SAS attendance or A and E admission was taken as an index date for cases and their respective controls.

The pharmacogenetic study of SU response in the GoDARTS study was described previously in detail.^8^ In the current study, we included 1770 patients with T2D who were incident users of SUs during the period 1994 to 2010. SU response was defined as the difference between pre‐treatment HbA1c and the minimum HbA1c measured within 1 to 18 months after initiation of SU. Linear regression was used to model the outcome of HbA1c reduction, using pre‐treatment HbA1c, age, sex, BMI, adherence, average daily dose, baseline gap (time between pre‐treatment HbA1c measurement and initiation of SU therapy) and a treatment group (SU prescribed as monotherapy or dual therapy: SU added to stable metformin treatment) as covariates.

In addition to HbA1c reduction, we used 2 other outcomes for efficacy, to take into account both HbA1c reduction and dosing of SU as composite measures of response: (1) the average prescribed dose of SU in the treatment period until minimum HbA1c was achieved and (2) a combined outcome of HbA1c reduction and prescribed SU daily dose, assessed as an aggregated Z‐score:

$$\text{Combined}\;Z‐\text{score}=\frac{\text{dose}–\text{mean}\;\left(\text{dose}\right)}{\mathrm{SD}\left(\text{dose}\right)}–\frac{\text{ΔHbA}1c–\text{mean}\;\left(\text{ΔHbA}1c\right)}{\mathrm{SD}\left(\text{ΔHbA}1c\right)}$$

Thus, a lower combined Z‐score indicates a better response to SU treatment. As patients were treated with different SU drugs, the prescribed daily dose was expressed as the percentage of maximum daily dose recommended by the British National Formulary.

The genotypes for CYP2C9*2, CYP2C9*3 and POR*28 variants were obtained from existing genome‐wide data.^13^ The frequencies of the minor alleles of CYP2C9*2, CYP2C9*3 and POR*28 were 12%, 7% and 27%, respectively. There was no deviation from Hardy–Weinberg equilibrium for any polymorphism. The genotypes for CYP2C9*2 and CYP2C9*3 were combined and analysed as the number of the CYP2C9*2 and CYP2C9*3 reduced‐function alleles: 0 (*1/*1), 1 (*1/*2, *1/*3) or 2 (*2/*2, *2/*3, *3/*3), in line with our previous study.^8^ An additive genetic model was used to assess the impact of the POR*28 variant, and the number of *CYP2C9* reduced‐function alleles, on hypoglycaemia and SU treatment outcomes.

Conditional logistic regression was used to analyse the effect of the genotypes on hypoglycaemia. The interaction between *POR* and *CYP2C9* combined genotypes was assessed by adding an interaction term to the regression model. In the analyses stratified by genotype, matching was broken and an unconditional logistic regression was used. All analyses were adjusted for age, sex, age of T2D diagnosis, BMI, creatinine and HbA1c. Statistical analysis was performed using SAS 9.3 software (SAS Institute Inc., Cary, North Carolina). The statistical significance level was set to *P* < .05.

## 3 RESULTS

The characteristics of patients with and without hypoglycaemia are shown in Table S2. Compared to control patients, patients with hypoglycaemia had lower BMI, lower HbA1c and higher creatinine levels. There was no difference in co‐treatment with medications known to be CYP2C9 inhibitors.

When assessed individually in the conditional logistic regression model, none of the variants showed association with hypoglycaemia (Table S3). Next, we explored the interaction between combined *CYP2C9* and *POR* genotypes by adding an interaction term to the model. The interaction between the 2 genes was significant (*P* = .007). In the analysis stratified by the *POR* genotypes, the number of *CYP2C9* deficient alleles increased the odds of hypoglycaemia nearly 3‐fold (OR, 2.81; 95% CI, 1.30‐6.09; *P* = .009) in the POR*1/*1 genotype group, whereas there was no effect in the POR*28 variant carriers (Table 1). However, when stratified by *CYP2C9* genotypes, the minor POR*28 allele showed no association with hypoglycaemia in either group (Table 1).

**Table 1:** Association of CYP2C9 and POR genotypes with SU‐induced hypoglycaemia: analyses stratified according to CYP2C9 and POR genotypes

Effect of *CYP2C9* deficient alleles on hypoglycaemia ‐ analysis stratified by *POR* genotype			
*POR* genotype	Cases/Controls	OR (95% CI)	*P*
POR *1/*1	29/151	2.81 (1.30‐6.09)	.009
POR *1/*28, *28/*28	33/133	0.70 (0.32‐1.57)	.390

Effect of POR*28 minor allele on hypoglycaemia ‐ analysis stratified by *CYP2C9* genotype			
*CYP2C9* genotype	Cases/Controls	OR (95% CI)	*P*
CYP2C9 *1/*1	35/180	1.58 (0.82‐3.03)	.174
CYP2C9 *2 or *3 carriers	27/104	0.58 (0.26‐1.28)	.177

We tested the association of genotypes with SU response assessed as 3 different endpoints. The characteristics of patients included in the analysis are presented in Table S4. The effects of variants on the 3 outcomes are shown in Table S5. The CYP2C9*3 variant (β, 0.145; SE, 0.063; *P* = .022) and the number of *CYP2C9* reduced‐function alleles (β, 0.098; SE, 0.041; *P* = .017) were associated with better SU response, assessed as HbA1c reduction, in line with our previous study.^8^ No variant showed any effect on prescribed SU dose, whilst POR*28 showed a marginal association with a lower combined Z‐score (β, −0.091; SE, 0.045; *P* = .043) and thus better response to SU treatment (Table S5).

There was a significant interaction between *CYP2C9* and *POR* genotypes, with both the prescribed dose (*P* = .004) and the combined Z‐score (*P* = .005) outcomes, but not with the simple reduction in HbA1c outcome (*P* = .484). In the stratified analysis, the reduced‐function *CYP2C9* alleles were associated with a lower combined Z‐score (greater response) only in patients with the POR *1/*1 genotype (β, −0.218; SE, 0.074; *P* = .003) (Table 2). On the other hand, the POR*28 allele showed association with better response only in CYP2C9*1/*1 carriers (β, −0.167; SE, 0.058; *P* = .004) (Table 2).

**Table 2:** Association of CYP2C9 and POR genotypes with SU response, assessed as the combined outcome of HbA1c reduction and prescribed daily dose (combined Z‐score): analyses stratified according to CYP2C9 and POR genotypes

Effect of *CYP2C9* deficient alleles on combined Z‐score ‐ analysis stratified by *POR* genotype				
*POR* genotype	N	Beta	SE	*P*
POR *1/*1	928	−0.218	0.074	.003
POR *1/*28, *28/*28	842	0.035	0.065	.588

Effect of POR*28 minor allele on combined Z‐score ‐ analysis stratified by *CYP2C9* genotype				
*CYP2C9* genotype	N	Beta	SE	P
CYP2C9 *1/*1	1130	−0.167	0.058	.004
CYP2C9 *2 or *3 carriers	640	0.059	0.071	.412

## 4 CONCLUSIONS

Although earlier studies demonstrated the influence of *CYP2C9* genetic polymorphisms on the metabolism and glycaemic response to SUs, data on the association between *CYP2C9* genotypes and SU‐induced hypoglycaemia are limited and inconsistent.^10^ The results of our study indicate that these discrepancies could be explained, at least in part, by the interaction between *CYP2C9* and *POR* genes. Although *CYP2C9* deficient alleles were not associated with hypoglycaemia in the entire group of patients, they increased the odds of hypoglycaemic events in patients carrying the POR*1/*1 genotype. Thus, we replicated a finding from the small study by Ragia et al.^11^ which showed an association between the CYP2C9*2 allele and higher risk of hypoglycaemia only in POR*1/*1 carriers.

Consistent with the effect on hypoglycaemia, the interaction between *CYP2C9* and *POR* genes also showed an association with the efficacy of SU treatment, assessed as a combined outcome of HbA1c reduction and prescribed SU daily dose. The *CYP2C9* deficient alleles were associated with a better treatment effect in POR*1/*1 carriers. This analysis of SU response in a much larger number of patients corroborates our findings for SU‐induced hypoglycaemia, which was explored in a smaller cohort.

In line with a previous study,^11^ the common POR*28 variant was not associated with hypoglycaemia in the entire group of patients, nor in subgroups stratified by the *CYP2C9* genotype. On the other hand, it showed an effect on better treatment response in CYP2C9*1/*1 carriers. This finding is in contrast to prior in vitro work, where POR*28 showed a modest increase in CYP2C9 activity, with flurbiprofen, diclofenac and tolbutamide as substrates.^12^ Our results, on the contrary, imply that POR*28 could possibly lead to decreased activity of the wild‐type CYP2C9, and thus to a higher concentration and an enhanced effect of SUs. Although this effect is possible, as it has been demonstrated that the impact of *POR* variants on CYP activities varies with the CYP isoform and the tested substrate,^14^ these intriguing results have not been replicated and have to be considered with caution.

In conclusion, we have shown that interaction between the *CYP2C9* and *POR* genes affects the risk of SU‐induced hypoglycaemia and the efficacy of SU treatment. These findings can contribute to unravelling the genetic causes of high inter‐individual differences in the efficacy and severe adverse effects of SU therapy, and may lead to safer treatment with these agents.

## Supporting information


**Table S1.** Structural and functional effects of CYP2C9*2, CYP2C9*3 and POR*28 variants.
**Table S2.** Characteristics of patients with and without hypoglycaemia.
**Table S3.** Association of CYP2C9*2, CYP2C9*3, combined CYP2C9 and POR genotypes with hypoglycaemia.
**Table S4.** Characteristics of patients included in the analysis of SU response.
**Table S5.** Effect of CYP2C9*2, CYP2C9*3, combined CYP2C9 and POR genotypes on SU response assessed as three different outcomes.
