# Supplementary material for: Interaction between variants in the CYP2C9 and POR genes and the risk of sulfonylurea‐induced hypoglycaemia: A GoDARTS Study
Source: Diabetes Obes Metab. 2017 Aug 25;20(1):211–4. doi: 10.1111/dom.13046 (PMC5724509; doi:10.1111/dom.13046)
Supplement: Supplementary file 1 — Table S1. Structural and functional effects of CYP2C9*2, CYP2C9*3 and POR*28 variants. Table S2. Characteristics of patients with and without hypoglycaemia. Table S3. Association of CYP2C9*2, CYP2C9*3, combined CYP2C9 and POR genotypes with hypoglycaemia. Table S4. Characteristics of patients included in the analysis of SU response. Table S5. Effect of CYP2C9*2, CYP2C9*3, combined CYP2C9 and POR genotypes on SU response assessed as three different outcomes. [file DOM-20-211-s001.docx]

**Supplementary Table 1 Structural and functional effects of *CYP2C9*2*, *CYP2C9*3* and *POR*28* variants.**

| **Polymorphism** | **dbSNP ID** | **Nucleotide**  **change** | **Amino acid**  **change** | **MAF^*^** | **Functional**  **effect** |
| --- | --- | --- | --- | --- | --- |
| ***CYP2C9*2*** | rs1799853 | c.430C>**T** | R144C | 0.12 | Decreased CYP2C9 activity |
| ***CYP2C9*3*** | rs1057910 | c.1075A>**C** | I359L | 0.07 | Markedly decreased CYP2C9 activity |
| ***POR*28*** | rs1057868 | c.1508C > **T** | A503V | 0.30 | Differential effect on CYP activities; Increased CYP2C9 activity^1^ |

Minor alleles are shown in bold. dbSNP, single nucleotide polymorphism database; ID, identification. ^*^Minor allele frequencies from 1000 Genome Project Phase 3 EUR population (www.ncbi.nlm.nih.gov/projects/SNP).

**Supplementary Table 2 Characteristics of patients with and without hypoglycaemia.**

|  | **Cases (n=69)** | **Controls (n=311)** | **OR (95% CI)** | ***P^*^*** |
| --- | --- | --- | --- | --- |
| Age (years) | 77.0 ± 8.4 | 76.4 ± 7.5 | 1.15 (0.73-1.81) | 0.554 |
| Age at diagnosis (years) | 64.1 ± 9.1 | 63.6 ± 8.2 | 1.06 (0.82-1.38) | 0.650 |
| Females/Males (Females %) | 32/37 (46.4%) | 139/172 (44.7%) | - | - |
| BMI (kg/m^2^) ^†^ | 28.5 ± 5.8 | 30.0 ± 5.5 | 0.94 (0.89-1.00) | 0.037 |
| HbA_1c_ (%)^‡^ | 7.0 ± 1.4 | 7.6 ± 1.3 | 0.69 (0.52-0.91) | 0.010 |
| Creatinine (µmol/l) | 106 (82-155) | 88 (71-107) | 1.02 (1.01-1.02) | <0.0001 |
| Other oral hypoglycaemic drug | 54 (78.3%) | 239 (76.9%) | 1.28 (0.64-2.57) | 0.491 |
| Co-treatment with CYP2C9 inhibitors^§^ | 5 (7.3%) | 17 (5.5%) | 1.33 (0.48-3.66) | 0.584 |
| Sulfonylurea drug  Glibenclamide  Gliclazide  Glimepiride  Glipizide | 2 (2.9%)  53 (76.8%)  4 (5.8%)  10 (14.5%) | 5 (1.6%)  254 (81.7%)  3 (1.0%)  49 (15.8%) | 2.12 (0.38-11.8)  0.74 (0.39-1.40)  5.13 (1.10-23.9)  0.93 (0.44-1.96) | 0.394  0.350  0.038  0.841 |

Data are presented as means ± SD, medians (interquartile range), or numbers (percentages). ^*^*P* value obtained with univariate conditional regression analysis. ^†^Data available for 68 cases and 305 controls. ^‡^Data available for 63 cases and 289 controls. ^§^Number of individuals concomitantly treated with CYP2C9 inhibitors, including fluconazole, miconazole, amiodarone, fluvastatin, isoniazid, sertraline, fluoxetine, co-trimaxozole, metronidazole, leflunomide, cimetidine and valproate.

**Supplementary Table 3** **Association of *CYP2C9*2*, *CYP2C9*3*, combined *CYP2C9* and *POR* genotypes with hypoglycaemia.**

| **Gene/ SNP** | **OR (95% CI)** | ***P*** |
| --- | --- | --- |
| *CYP2C9*2* | 1.40 (0.75-2.61) | 0.289 |
| *CYP2C9*3* | 0.91 (0.36-2.33) | 0.848 |
| *CYP2C9* deficient alleles^*^ | 1.26 (0.72-2.18) | 0.420 |
| *POR*28* | 1.04 (0.63-1.72) | 0.885 |

^*^Combined genotype for CYP2C9 - number of deficient

alleles (*2 or *3). Conditional logistic regression analysis

included 62 cases and 284 controls.

**Supplementary Table 4 Characteristics of patients included in the analysis of SU response.**

| Number of participants | 1,770 |
| --- | --- |
| Age (years) | 62.8 ± 11.0 |
| Females/Males (Females %) | 770/1,000 (43.5%) |
| BMI (kg/m^2^) | 30.6 ± 5.6 |
| Daily dose (equivalent dose, %) | 25.0 (16.7-37.5) |
| Adherence (%)^*^ | 80.3 ± 16.0 |
| Baseline gap (days)^†^ | 7 (2-21) |
| Pre-treatment HbA_1c_ (%) | 9.1 ± 1.4 |
| On-treatment HbA_1c_ (%) | 7.0 ± 1.1 |
| SU monotherapy | 821 (46.4%) |
| Co-treatment with CYP2C9 inhibitors^‡^ | 134 (7.6%) |
| Sulfonylurea drug  Glibenclamide  Gliclazide  Glimepiride  Glipizide | 32 (1.8%)  1,449 (81.9%)  38 (2.1%)  251 (14.2%) |

Data are presented as means ± SD, medians (interquartile range), or numbers (percentages). ^*^Adherence was calculated from the drug dispensing records. ^†^Time between pre-treatment HbA_1c_ measurement and the start of SU therapy. ^‡^Number of individuals concomitantly treated with CYP2C9 inhibitors, including fluconazole, miconazole, amiodarone, fluvastatin, sertraline, fluoxetine, co-trimaxozole, metronidazole, tamoxifen, cimetidine and valproate.

**Supplementary Table 5** **Effect of *CYP2C9*2*, *CYP2C9*3*, combined *CYP2C9* and *POR* genotypes on SU response assessed as three different outcomes.**

|  | **Reduction in HbA_1c_** | | | **Daily dose** | | | **Combined Z-score**^†^ | | |
| --- | --- | --- | --- | --- | --- | --- | --- | --- | --- |
| **Gene/ SNP** | **Beta** | **SE** | ***P*** | **Beta** | **SE** | ***P*** | **Beta** | **SE** | ***P*** |
| *CYP2C9*2* | 0.053 | 0.049 | 0.278 | -0.003 | 0.010 | 0.746 | -0.051 | 0.059 | 0.391 |
| *CYP2C9*3* | 0.145 | 0.063 | 0.022 | -0.008 | 0.012 | 0.533 | -0.134 | 0.076 | 0.078 |
| *CYP2C9* deficient alleles^*^ | 0.098 | 0.041 | 0.017 | -0.005 | 0.008 | 0.500 | -0.091 | 0.049 | 0.063 |
| *POR*28* | 0.061 | 0.037 | 0.103 | -0.010 | 0.007 | 0.176 | -0.091 | 0.045 | 0.043 |

Linear regression analysis included 1,770 patients. ^*^Combined genotype for CYP2C9 - number of deficient alleles (*2 or *3). ^†^Aggregated Z-score for combined outcome of HbA_1c_ reduction and prescribed SU dose.

**References**

1. Subramanian M, Agrawal V, Sandee D, Tam HK, Miller WL, Tracy TS. Effect of P450 oxidoreductase variants on the metabolism of model substrates mediated by CYP2C9.1, CYP2C9.2, and CYP2C9.3. Pharmacogenetics and genomics. 2012;22:590-597.
